# Dissecting the Neuronal Contributions of the Lipid Regulator NHR-49 Function in Lifespan and Behavior in *C. elegans*

**DOI:** 10.3390/life13122346

**Published:** 2023-12-15

**Authors:** Saebom Kwon, Kyu-Sang Park, Kyoung-hye Yoon

**Affiliations:** 1 Department of Physiology, Yonsei University Wonju College of Medicine, Wonju 26426, Republic of Korea; saebom0504@naver.com; 2 Mitohormesis Research Center, Yonsei University Wonju College of Medicine, Wonju 26426, Republic of Korea; 3 Department of Global Medical Science, Yonsei University of Wonju College of Medicine, 20 Ilsan-ro, Wonju 26426, Republic of Korea

**Keywords:** *Caenorhabditis elegans*, NHR-49, lipid metabolism, neuron, behavior

## Abstract

Although the importance of lipid homeostasis in neuronal function is undisputed, how they are regulated within neurons to support their unique function is an area of active study. NHR-49 is a nuclear hormone receptor functionally similar to PPARα, and a major lipid regulator in *C. elegans*. Although expressed in most tissues, little is known about its roles outside the intestine, the main metabolic organ of *C. elegans*. Here, using tissue- and neuron-type-specific transgenic strains, we examined the contribution of neuronal NHR-49 to cell-autonomous and non-autonomous *nhr-49* mutant phenotypes. We examined lifespan, brood size, early egg-laying, and reduced locomotion on food. We found that lifespan and brood size could be rescued by neuronal NHR-49, and that NHR-49 in cholinergic and serotonergic neurons is sufficient to restore lifespan. For behavioral phenotypes, NHR-49 in serotonergic neurons was sufficient to control egg-laying, whereas no single tissue or neuron type was able to rescue the enhanced on-food slowing behavior. Our study shows that NHR-49 can function in single neuron types to regulate *C. elegans* physiology and behavior, and provides a platform to further investigate how lipid metabolism in neurons impact neuronal function and overall health of the organism.

## 1. Introduction

Many instances of neurological and neurodegenerative disorders involve various forms of underlying metabolic dysfunctions, including lipid dysregulation [1]. However, precise understanding of the upstream causes that lead to such effects remain unclear. In addition, studies of lipid metabolism in neurons are made difficult in most systems by the fact that they work closely with the glia, making it difficult to disentangle one’s function from another. The free-living nematode *Caenorhabditis elegans* provides an ideal in vivo model to study the effect of lipid metabolism with a simple nervous system composed of all major neuron subtypes, such as glutamatergic, GABAergic, cholinergic, serotonergic, and dopaminergic, and includes only a few glial cells.

NHR-49 is one of the many nuclear hormone receptors expressed in *C. elegans*. *C. elegans* possesses a total of 284 nuclear hormone receptors in its genome, but the function of most remains elusive [2]. NHR-49 is one of the well-studied nuclear hormone receptors, first discovered for its role in the *C. elegans* fasting response, where animals metabolize its lipid stores to produce energy [3]. NHR-49 regulates expression of genes involved in fatty acid β-oxidation and desaturation [4]. Lack of *nhr-49* resulted in decreased survival during starvation [3]. Aside from the fasting response, *nhr-49* mutants display additional pleiotropic defects, such as shorter lifespan, impaired proteostasis, and vulnerability to various stressors such as oxidative stress, pathogenic infection, and hypoxia [5,6,7,8,9,10]. Such defects demonstrate the importance of lipid homeostasis in a wide variety of processes.

Although the intestine is the main metabolic organ and site of lipid storage in *C. elegans*, NHR-49 is expressed in most tissues in the animal, including neurons. Neurons generally have limited lipid catabolic capabilities due to their vulnerability to ROS [11]. They do not store fats, and it has been shown in mammalian and fly nervous systems that they instead receive lipid-derived lactate from astrocytes or glia as an energy source [11,12]. Considering this, it is unclear what role a transcription factor that mainly regulates fatty acid β-oxidation genes would play in neurons. In light of recent studies that have shown contributions of neuronal NHR-49 to lifespan and immunity [8,13], further study of the role of the lipid regulator in neurons may improve our understanding of how neuronal lipid metabolism contributes to cell-autonomous and non-autonomous functions. Similar to the neuronal subtypes in the vertebrate nervous system, *C. elegans* neurons express neurotransmitters and neuromodulators such as acetylcholine, GABA, serotonin, and dopamine. Therefore, studies in contributions of specific neuronal subtypes to lipid-mediated inter-tissue signaling in *C. elegans* may be significant and applicable to mammalian systems. In addition, since phenotypes such as lifespan and immunity are complex phenotypes with many contributing factors, it is difficult to precisely define the mechanism of neuronal NHR-49. Therefore, identification of more specific neuronal and behavioral phenotypes of *nhr-49* may provide a better system to study the precise roles and mechanism of lipid metabolism in neurons, which may in turn reveal how it supports the overall health of the animal. 

Here, we used tissue- and neuron-type-specific transgenic rescue strains of *nhr-49* to investigate the contribution of neuronal NHR-49 to various *nhr-49* phenotypes. The phenotypes we observed are lifespan, a previously established phenotype, as well as brood size, egg-laying, and on-food slowing behavior, which are previously unreported phenotypes of *nhr-49* described here for the first time. We found that neuronal NHR-49 can cell-non-autonomously contribute to both lifespan and brood size, and more specifically, NHR-49 in cholinergic and serotonergic neurons are sufficient to prolong lifespan in the mutants. For egg-laying, NHR-49 in serotonergic neurons was sufficient to restore the defect, likely demonstrating a direct cell-autonomous effect of NHR-49 in neuronal function. On the contrary, enhanced on-food slowing behavior could not be rescued by NHR-49 in any single tissue, suggesting it is likely mediated by signaling between multiple tissues with functional NHR-49. 

## 2. Materials and Methods

### 2.1. C. elegans Growth and Maintenance

All strains used for the assay are listed in Table S2. Strains were grown in nematode growth media (NGM) plates seeded with OP50 *E. coli* bacteria as previously described. Strains were maintained at 20 °C.

### 2.2. Plasmid Constructs and Generation of Transgenic Lines

Transgenic strains generated for the study are listed in Table S2. All primers used in this study are listed in Table S3. All neuronal-promoter-driven *nhr-49* plasmids were constructed using the pPD95.75 plasmid as backbone. *nhr-49c* was cloned from *C. elegans* cDNA. This particular isoform was chosen due to having the longest coding region and because it had been previously used by other labs for transgenic rescue strains [13]. Each segment of the plasmid, promoter, *nhr-49*, and the SL2 trans-splicing sequence was ligated into the plasmid backbone using the restriction enzyme sites indicated in the primer sequences in Table S3. To subclone *tph-1* and *dat-1* promoters, which were flanked by NotI and SmaI sites, we created a NotI site in the plasmid by inserting a custom-designed multiple cloning site using annealed single strand oligos with appropriate overhangs (MCS, Table S3). For muscle-specific expression of *nhr-49*, the same *nhr-49* insert was inserted into the *myo-3* promoter containing plasmid, which was a gift from Junho Lee. *nhr-49(nr2041)* mutant strains were injected with each promoter plasmid (20 ng/μL) and co-injection marker *unc-122p*::mCherry (30 ng/μL), *unc-122p*::gfp or *myo-2p*::mCherry (5 ng/μL) co-injection marker. Promoters used for tissue- and neuron-type-specific expression are as follows: 3.5 kb *rgef-1* promoter for pan-neuronal expression [14], 3.2 kb *unc-17* promoter for cholinergic neurons [15], 2.4 kb *eat-4* promoter for glutamatergic neurons [16], 1.75 kb *tph-1* promoter for serotonergic neurons [17], 716 bp *dat-1* promoter for dopaminergic neurons [18], and 1.8 kb *unc-25* promoter for GABAergic neurons [19]. 

### 2.3. Lifespan Assays

Worms were synchronized by bleach and grown until L4 larval stage. Approximately 20–25 worms were placed in each NGM plate containing 120 μM FuDR (5-Fluoro-2′-deoxyuridine) to prevent eggs from hatching. Worms were counted every 2–3 days until all worms were dead. Three trials were conducted for each strain.

### 2.4. Scoring Embryonic Stage of Freshly Laid Eggs

A previously established protocol was followed [20]. In short, 5 adult worms were placed on seeded NGM plates and left for an hour, after which they were removed. Immediately, eggs laid on plates were observed under a microscope to determine their embryonic stage. Brood size was determined by placing a single L4 worm in a seeded NGM plate. Worms were moved to a new plate daily until they stopped laying new eggs. Hatched worms were counted in each plate after 2–3 days to assess total number of eggs laid by the worm.

### 2.5. Measuring Worm Speed and Path Angle

Worm speed was measured following a previously established protocol with some modifications [21]. Between 7–9 worms were placed in a bacterial lawn, were left to acclimate for 4 min, then their movement was recorded for 4 min at two frames per second. Three trials were conducted for each strain, with the exception of the muscle-specific rescue strain. For measuring off-food speed, about 20 worms were picked and placed in an unseeded agar plate to remove any food adhered to the body, then moved to a fresh assay plate where movement was recorded for 4 min. Average speed of worms was determined using the wrmTrck plugin in Fiji (https://imagej.net/Fiji, accessed on 2 May 2023) [22]. Worm location within the plate was tracked using the “Analyze Particle” function in Fiji. The coordinates provided in the results table were used to plot its location relative to time using Matplotlib in Python. Path angle was measured by first finding the coordinates of each worm using the MTrack2 plugin in Fiji, then extracting coordinates at 10-s intervals to determine path angle with a Python script.

### 2.6. Statistical Analysis

Statistical analysis for worm lifespan was conducted using OASIS 2 Kaplan–Meier analysis and Log-Rank test [23]. Statistical analysis for worm velocity was compared using unpaired *t*-test. Brood size was compared using one-way ANOVA with Dunnett’s post hoc test comparison to *nhr-49*. Statistical analysis for embryonic stage distribution of fresh laid eggs was determined by Wilcoxon Mann–Whitney rank sum test in comparison to *nhr-49*. Statistical significance is indicated by asterisks as follows: *p* < 0.05 (*), *p* < 0.01 (**), *p* < 0.001 (***), and *p* < 0.0001 (****).

## 3. Results

### 3.1. NHR-49 in Select Neuron Types Contribute to Lifespan

**Figure 1:**
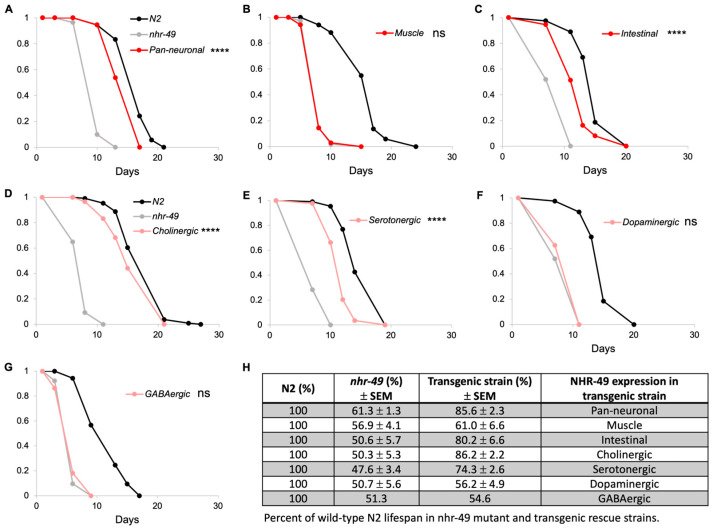
NHR-49 in select neuron types contributes to lifespan. (**A**–**C**) Lifespan of pan-neuronal (**A**), muscle (**B**), and intestinal (**C**) transgenic rescue strains in comparison to wild-type *N2* and *nhr-49* mutant strains. (**D**–**G**) Lifespan of neuron-type specific rescue strains. Each graph represents one trial of lifespan assay, since lifespan can vary significantly between trials. (**H**) Summary of all trials indicated in percentages of mean lifespan compared to N2 wild-type. Mean lifespan of *nhr-49* mutant and transgenic strains for each trial was normalized against that of wild-type N2. Values shown represent average of the normalized values and standard error of mean. Three trials were conducted with the exception of the GABAergic rescue strain. Complete data and statistical analyses for all trials are presented in Table S1. Asterisks indicate *p* value (**** *p* < 0.0001, ns: not significant).

Mutants of *nhr-49* display short lifespan compared to wild-type *C. elegans*. Interestingly, previous studies have shown that transgenic expression of NHR-49 in either neuronal or intestinal tissue restores most of the short lifespan defect in *nhr-49* mutants [8,13]. We confirmed these results by generating a pan-neuronal rescue strain in *nhr-49* mutants using the *rgef-1* promoter, a pan-neuronal promoter with no observable intestinal leakiness often seen in other neuronal promoters [24,25,26] (Figure 1A,H and Figure S1; Table S1). We also generated a muscle-specific rescue transgenic strain and found that expression of NHR-49 in the muscle of *nhr-49* mutants had no effect on lifespan (Figure 1B,H). The intestine-specific rescue showed restored lifespan as reported previously [13] (Figure 1C,H). 

If NHR-49 in neurons alone is sufficient to restore normal lifespan, would any specific neuron type be contributing to this? *C. elegans* neurons express neurotransmitters and neuromodulators such as acetylcholine, GABA, serotonin, and dopamine that regulate motor control, behaviors, and various neuronal functions (Table 1). To examine if NHR-49 expression in specific subsets of neurons is responsible for lifespan, we generated strains in which *nhr-49* was restored in either cholinergic, GABAergic, serotonergic, or dopaminergic neurons. Interestingly, we found that there was indeed a neuron-type specificity to the contribution of NHR-49 to lifespan: whereas NHR-49 expression in dopaminergic and GABAergic had no effect (Figure 1F–H; Table S1), NHR-49 expression in cholinergic and serotonergic showed recovery of lifespan to 86.2% and 74.3%, respectively, that appeared similar to the lifespan recovery of 85.6% in the pan-neuronal rescue strain (Figure 1D,E,H; Table S1). Thus, we show that not all neurons equally contribute to lifespan, but NHR-49 in certain neuron types contributes more than others. 

**Table 1:** Neuron subtypes in *C. elegans* according to neurotransmitters or neuromodulators.

Promoter	Neuron Type	# of Neurons	Associated Function
dat-1	Dopaminergic	8	Touch sensation [27], locomotion [28], foraging behavior [27,29], learning [30]
tph-1	Serotonergic	3	Egg-laying [31], foraging behavior [27], pharyngeal pumping [32]
unc-25	GABAergic	26	Locomotion [33], immunity [34]
unc-17	Cholinergic	160	Locomotion, male mating, egg-laying [35]
eat-4	Glutamatergic	79	Sensory signaling [36]

### 3.2. NHR-49 in Neurons Contributes to Egg Production

**Figure 2:**
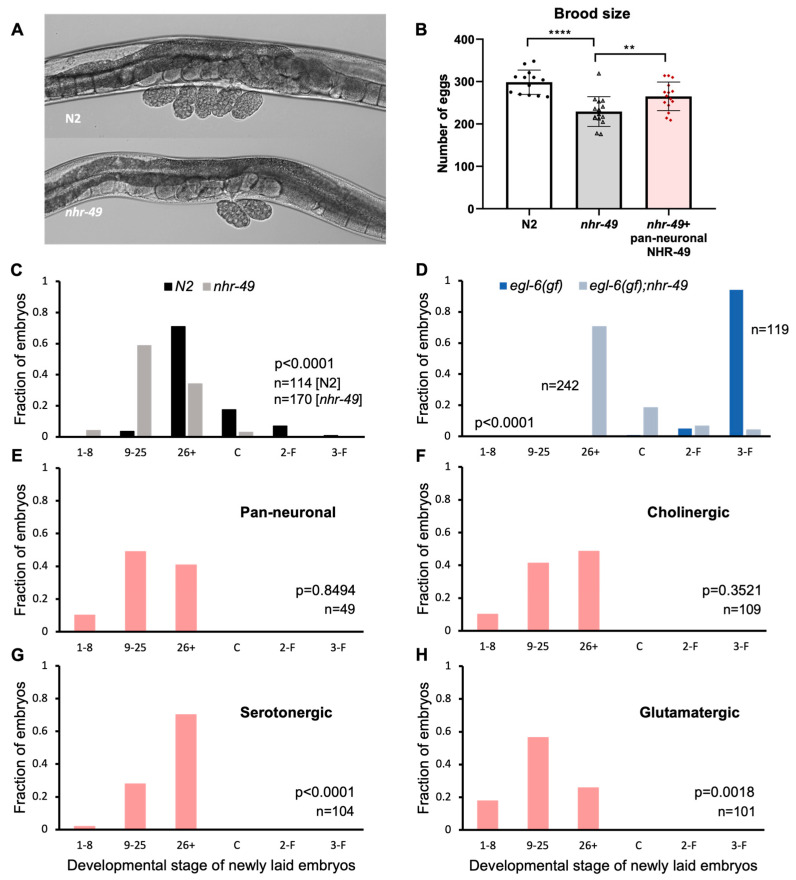
NHR-49 in select neuron types contribute to egg-laying. (**A**) Images of eggs carried by *N2* and *nhr-49*. Recently laid eggs collected around the vulva in *N2* show embryos in the 9–25 and 26+ stages, and two 9–25 stage eggs for *nhr-49* mutants. (**B**) Total number of eggs laid by *N2*, *nhr-49*, and pan-neuronal NHR-49 transgenic. Asterisks indicate *p* value (** *p* < 0.01, **** *p* < 0.0001) (**C**) Embryonic stages of freshly laid eggs in *N2* and *nhr-49* mutants. (**D**) Embryonic stages of freshly laid eggs in *egl-6(gf)* and *nhr-49;egl-6(gf).* (**E**–**H**) Embryonic stages of freshly laid eggs in pan-neuronal (**E**), cholinergic (**F**), serotonergic (**G**), and glutamatergic (**H**) transgenic rescue strains. *p* values determined by the Wilcoxon Mann–Whitney rank sum test are indicated for each graph (**C**–**H**).

*C. elegans* hermaphrodites begin laying eggs a few hours after becoming adults and continue to lay at a regular rate for a few days, resulting in about 300 laid eggs. Egg-laying behavior in the hermaphrodite is tightly regulated by vulval and uterine muscles innervated by motor neurons that express the neurotransmitters acetylcholine and serotonin (Table 1) [37]. Accumulation of embryos in the uteri induces motor neuron activity resulting in egg-laying [38]. While working with the mutants, we noticed that *nhr-49* mutants carried a smaller number of eggs in the uterus. Wild-type N2 worms carry 10–15 eggs in the body, but *nhr-49* adults tend to carry less than 10 (Figure 2A). Although easily recognizable, the phenotype has never been reported or closely studied. The number of eggs carried by the adult worms may indicate a difference in the rate of input—oocyte maturation and fertilization, or the rate of output—how soon the eggs are laid. We investigated both possibilities to see which are affected by NHR-49.

To see if there is a difference in brood size between N2 and *nhr-49* mutants, we counted the total number of eggs laid during the lifetime of the worms. We found that *nhr-49* mutants did indeed lay fewer eggs—an average of 230 eggs compared to almost 300 laid by wild-type worms (Figure 2B). Interestingly, we found that pan-neuronal rescue of *nhr-49* was sufficient to bring the total eggs closer to wild-type levels, laying an average of 265 eggs. This indicates that NHR-49 function in the neurons can contribute to brood size (Figure 2B). 

### 3.3. NHR-49 in Serotonergic Neurons Contributes to Egg Laying

Next, to see if the absence of *nhr-49* affected egg-laying behavior, we used a previously established protocol to score embryonic stages of freshly laid eggs [20]. The mitotic divisions and morphological changes of *C. elegans* embryos can be easily observed through its clear outer shell, which allowed us to sort the egg stages into six categories: 1–8 cell, 9–25 cell, 26+ cell, comma, 2-fold, and 3-fold stages. The first two stages are easily recognizable by the number of cells in the embryo. At 26+ cell stage, the embryo is a cluster of innumerable cells and undergoes gastrulation. Afterwards an indentation forms at the side of the embryo, which is called the comma stage, then the worm shape begins to take hold, with the elongated shape curled into 2-fold then 3-fold in the tight space within the egg shell. Wild-type *N2* hermaphrodites laid eggs that are mostly in the 26+ embryonic stage, which is consistent with previous studies [20] (Figure 2C). On the other hand, more than half of the eggs laid by *nhr-49* mutants were in the 9–25 stage, showing a clear shift to earlier stage embryos (Figure 2C).

Vulva muscles receive input from the egg-laying motor circuit, which includes VC cholinergic motor neurons and the HSN motor neuron that is both serotonergic and cholinergic [37]. Is NHR-49 acting through the neurons to control egg-laying? To strengthen the possibility that the egg laying phenotype of *nhr-49* mutants is due to altered activity of the neuronal circuit, we took advantage of the *egl-6* gain-of-function mutant strain, which exhibits high egg retention due to suppression of HSN motor neuron activity [20]. Genes that counteract the HSN inhibition can be tested through monitoring egg-laying in the double mutant with the *egl-6(gf)* allele. 

When we observed egg-laying in the *egl-6(gf)* background, there was a much wider difference between *nhr-49* wild-type and mutant allele (Figure 2D). Whereas eggs laid by *egl-6(gf)* mutants were mostly in the 3-fold stage, the last embryonic stage before hatching, eggs from *nhr-49;egl-6(gf)* double mutants were mostly in the 26+ stage. The fact that *nhr-49* mutants relieved egg retention caused by the suppression of a very specific neuron, indicated that the egg-laying phenotype of *nhr-49* mutants is likely due to a neuronal defect. 

To see if neuronal NHR-49 can restore normal egg-laying, we conducted the same assay on tissue-specific transgenic rescue strains. When eggs were scored in the pan-neuronal rescue, very little difference was observed compared to the mutant (Figure 2E). NHR-49 expression in cholinergic neurons resulted in a slight shift toward later embryonic stages, but was not statistically significant (Figure 2F). However, NHR-49 expression in serotonergic neurons resulted in a much larger shift towards later stage eggs, bringing the distribution closer to which is seen in wild-type worms (Figure 2G). Interestingly, NHR-49 in glutamatergic neurons resulted in even earlier stage eggs than in the mutant, indicating that restoring NHR-49 in the glutamatergic neurons exacerbated the early egg-laying phenotype (Figure 2H). This may explain the lack of improvement in the pan-neuronal rescue strain, as different neuron types affect egg-laying in different directions. Our results show that functional NHR-49 in the serotonergic neurons contributes to their neuronal function to mediate egg-laying. 

### 3.4. Reduced On-Food Speed in nhr-49 Mutants Is Not Due to NHR-49 in Any Single Tissue

**Figure 3:**
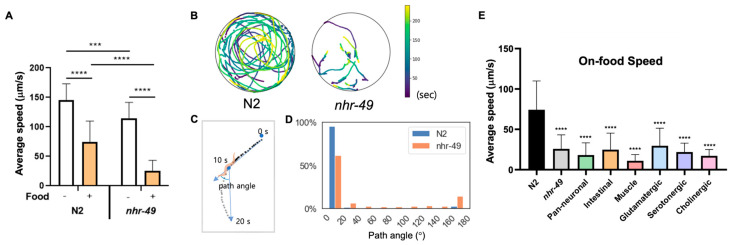
Reduced on-food speed in *nhr-49* mutants is not due to NHR-49 in any single tissue. (**A**) Average speed of N2 and *nhr-49* worms with and without food. (**B**) Trajectories of nine N2 and eight *nhr-49* worms on bacterial lawn. Color indicates the time during the 240 s when worms were found in the given location. Video recordings used for analysis can be seen in Videos S1 and S2, respectively. (**C**) Sample worm trajectory for 20 s and definition of path angle. Each dot indicates location of a worm in each frame (2 frame/s). Path angle was determined using worm locations at 10-s intervals. (**D**) Histogram of path angle frequency in each genotype. (**E**) Average speed of N2, *nhr-49*, and each tissue and neuron type transgenic rescue strains. Asterisks indicate *p* value (*** *p* < 0.001, **** *p* < 0.0001).

Lastly, we noticed that while *nhr-49* mutants display no overt movement defects, they traveled very little when placed in the bacterial lawn, a trait easily observable by the tracks left by the worms (Videos S1 and S2). *C. elegans* employ various foraging and exploratory behaviors to increase their chance of finding food and staying on food. The speed of movement, and size of the area explored, and distance traveled are dependent on factors such as time the animal has been away from food, internal nutrient levels, quality of food, as well as past nutritional experience of the worm [27,39,40,41]. When *C. elegans* approach a bacterial lawn, they slow down their speed as they enter the bacterial food, which is called the basal slowing response [27]. This is thought to allow worms to stay where food is plentiful, and requires dopaminergic signaling and mechanical sensing of bacteria [27]. We found that N2 animals display basal slowing response reducing their movement speed by approximately 50% on food (Figure 3A). *nhr-49* mutants show slightly reduced speed off food compared to wild-type, although their motility and gait overall appeared normal (Figure 3A, Videos S3 and S4). However, on food, *nhr-49* mutants displayed around 80% decreased velocity, a drastic reduction compared to basal slowing response in wild-type animals (Figure 3A).

*C. elegans* displays two modes of movement behavior on food called roaming and dwelling. Roaming behavior is characterized by relatively high movement speed with little curvature, whereas dwelling is defined by much slower speed with curved trajectories and frequent reversal of movement [41,42,43]. Differences in on-food movement are seen particularly between well-fed and starved animals: well-fed *C. elegans* display both roaming and dwelling behaviors, whereas starved worms exclusively show dwelling behavior [43]. To characterize whether the acute change in on-food speed in *nhr-49* is consistent with dwelling behavior, we recorded by video the movement trajectories of N2 and *nhr-49* mutants on food (Figure 3B). Overall, we found that wild-type worms moved about, exploring the whole area of the lawn, whereas movement of *nhr-49* mutants was much more subdued, with some animals hardly moving during the 4 min of recording (Figure 3B). In addition, when we analyzed the path angle of their movements, N2 worms mostly traveled with little curvature, whereas *nhr-49* movement contained more angle bends and reversals, which are indicated by path angles close to 180° (Figure 3C,D). On the contrary, when off-food, very little difference was observed in the distribution of path angles (Figure S2A,B). This suggests that the enhanced on-food slowing observed in *nhr-49* mutants is not only due to a general decrease in motility, but a heightened reaction to the presence of food. Thus, *nhr-49* mutants display altered on-food behavior, with much decreased speed and increased curvature and changes in direction compared to wild-type.

To find out whether NHR-49 in a specific tissue was responsible for the enhanced slowing on food, we tested on-food speed of the tissue-specific transgenic rescue strains. We tested pan-neuronal as well as intestinal rescue strains, since *nhr-49* could be involved in the internal nutrition signal originating from the intestine. Surprisingly, none of the tissue-specific strains tested—neuronal, intestinal, and muscle-specific—restored the on-food speed back to wild-type levels (Figure 3E). In fact, the pan-neuronal rescue and muscle-specific rescue strains showed a tendency to suppress on-food speed even further. Likewise, none of the neuron-type specific rescues of *nhr-49* showed improvement in on-food speed (Figure 3E). This may indicate either the enhanced basal slowing is mediated by yet another tissue that we have not yet tested, such as the hypodermis, or that it cannot be attributed to NHR-49 function in any single tissue. In the latter case, it may indicate that the behavior likely involves NHR-49 function in multiple tissues.

## 4. Discussion

The importance of lipid regulation in metabolism, health, and lifespan has been well established. Indeed, the importance of the lipid regulator NHR-49 in fat regulation, lifespan, and health has been substantiated by numerous studies in *C. elegans* [3,5,6,7,8,9,10,44]. However, the role that lipid regulation plays in the nervous system and behavior is not as clear. 

Studies of lipid regulation in *C. elegans* inevitably focus on the intestine, since it is the main metabolic organ that stores and processes fat. However, a few *nhr-49* mutant phenotypes, such as reduced lifespan and immunity, have been shown to improve upon restoring NHR-49 in the neurons, suggesting that NHR-49 contributes to the overall health of the worm through its lipid regulator function in neurons [8,13]. Studies of how lipid utilization and metabolism within neurons contribute to these phenotypes, though, have been lacking. In light of reports that various neurodegenerative diseases are often found to occur together with metabolic dysfunction [45,46], understanding how metabolism in neurons affects the rest of the body and vice versa will help illuminate the complex interplay between the nervous system and the rest of the body. *C. elegans* provides an ideal model to study the role of lipid regulators in neurons, with its simple nervous system of 302 neurons and only a small number of glia. Because phenotypes such as lifespan and immunity are complex phenotypes with many contributing factors, identification of more specific neuronal and behavioral phenotypes of *nhr-49* may provide a better system to study the immediate roles and mechanism of lipid metabolism in neurons, which in turn may help reveal the mechanism of its cell-non-autonomous role. 

Here, we characterized various known, as well as newly described, phenotypes of *nhr-49* mutants using tissue-specific transgenic strains. We confirmed previous reports that neuronal NHR-49 can restore lifespan, and further narrowed down the neurons responsible to cholinergic and serotonergic neurons. We also identified additional *nhr-49* mutant phenotypes, such as reduced brood size, early egg-laying, and slow on-food speed. Reduced brood size was also restored closer to wild-type levels by expression of neuronal NHR-49. For early egg-laying, pan-neuronal NHR-49 had very little effect, but serotonergic neuron-specific rescue significantly restored the early egg-laying defect. Lastly, *nhr-49* mutants displayed dramatically reduced on-food speed, but no single tissue rescued the phenotype.

Lifespan and brood size are complex phenotypes with many contributing factors. Instead of all neurons each contributing to the health of the animal, we found that NHR-49 in specific neuron types, cholinergic and serotonergic neurons, are sufficient to mediate restored lifespan, while other neuron types had no effect. This may indicate either that certain neuron types are more involved in influencing lifespan, or that NHR-49 serves an especially important role in those neurons to maintain their function. Further studies to narrow down the neurons responsible may help reveal the underlying mechanism.

Brood size in *C. elegans* is tightly linked to nutrient availability or past experience of dauer [47]. In general, brood size in unmated hermaphrodites is limited by the number of sperm, whose production occurs only during the L4 larval stage [48]. However, because both sperm and oocytes differentiate from the same pool of germline cells, general decrease in germline proliferation may be a better explanation for the reduced brood size seen in *nhr-49* mutants, rather than a specific defect in sperm development. The fact that pan-neuronal NHR-49 can rescue this phenotype underscores the contribution of neuronal NHR-49 to the overall health of the animal. 

The main neuron that governs vulval muscle contraction and egg-laying is the serotonergic HSN motor neuron. The HSN neuron cycles through a series of active and quiescent activity states, controlling the rate of egg-laying [37]. EGL-6 is an inhibitory GPCR, whose gain-of-function mutation results in suppression of HSN activity, leading to the characteristic accumulation of late-stage eggs in the mutants [20]. Mutations in the G-protein signaling pathway or inward rectifying potassium channel that works downstream of EGL-6 can suppress the egg retention phenotype of *egl-6(gf)* mutants [49]. The fact that *nhr-49;egl-6(gf)* double mutants can also relieve the *egl-6(gf)* egg retention phenotype strongly suggests that the absence of *nhr-49* results in increased neuronal activity in HSN. Consistently, restoring NHR-49 expression selectively in the serotonergic neurons was sufficient to cause *nhr-49* mutant worms to hold eggs until later embryonic stages. There are three serotonergic neurons in *C. elegans*: ADF, NSM, and HSN. Among these, only HSN is directly involved in egg-laying. Our data provide strong evidence that NHR-49 may directly impact HSN activity. Further investigation into the effect of NHR-49 and relevant downstream targets in HSN may lead to better understanding of how lipid metabolism affects neuronal activity and function.

*C. elegans* motor neurons control forward and backward locomotion to allow the worm to move sinusoidally to search for sources of bacterial food [50]. Movement of worms in bacterial lawns has been studied in *C. elegans* in relation to foraging behavior and food sensing, which are affected by external cues such as food concentration [43], as well as internal states such as hunger, and the nutritional quality of the food [41,51]. In addition to the general slowing down of speed on food, worms alternate between states of dwelling and roaming. Longer recording of worm movement is needed to determine whether their switch between dwelling and roaming is impacted, but the pattern of movement of *nhr-49* on food may suggest increased dwelling behavior as well as reduced speed. Such reduced speed and increased dwelling are often observed in starved worms [27]. The fact that *nhr-49* mutants display behavior similar to starved worms is consistent with having defective lipid metabolism. Surprisingly, none of the tissue-specific transgenic strains rescued the enhanced slowing response. This may indicate that the behavior requires NHR-49 in yet another tissue that we have not yet tested, such as the hypodermis, or that it requires NHR-49 in multiple tissues. Since sensing of starvation and nutrition is a whole-body response, it may require NHR-49 function in multiple tissues acting together, in order to assess organismal nutritional state and carry out the correct behavior.

Several studies using *C. elegans* have reported cell-non-autonomous effects from neurons that result in improvement in whole body proteostasis, stress resistance, and longevity [52,53,54,55]. In addition, Zullo et al. reported that inhibition of neural excitation and neurotransmission during aging increases longevity in both *C. elegans* and humans [56]. These studies demonstrate how brain function is intricately tied to the health and homeostasis of the whole body. What is still unclear, however, is how these cellular perturbations occurring in the neurons result in such whole-body effects. Although some specific signals are known, many are still unidentified [57,58,59]. Whether neuronal NHR-49 impacts lifespan and pathogenic infection via similar signals is yet to be determined. Recently, Savini et al. reported that neuronal NHR-49 is required for neuropeptide gene induction that mediates longevity in response to lysosomal lipolysis in the intestine [60], suggesting that NHR-49 may support neuropeptide signaling. Elucidating the nature of the cell-nonautonomous functions of the nervous system will enhance our understanding the intricate relationship between the brain and whole body.

## Data Availability

The data presented in this study are available upon request.

## Supplementary Materials

The following supporting information can be downloaded at: https://www.mdpi.com/article/10.3390/life13122346/s1, Figure S1: Neuron-type GFP expression in each transgenic strain (closed arrowhead). Open arrowheads indicate co-injection marker fluorescence; Figure S2: Trajectories and path angles of N2 and *nhr-49* worms off-food; Table S1: Summary of all lifespan assays; Table S2: Strains used in this study; Table S3: Primers used for this study; Video S1: On-food movement of wild-type N2 worms; Video S2: On-food movement of *nhr-49* mutants; Video S3: Off-food movement of wild-type N2 worms; Video S4: Off-food movement of *nhr-49* mutants.
