# Supplementary material for: Dissecting the Neuronal Contributions of the Lipid Regulator NHR-49 Function in Lifespan and Behavior in C. elegans
Source: Life (Basel). 2023 Dec 15;13(12):2346. doi: 10.3390/life13122346 (PMC10744624; doi:10.3390/life13122346)
Supplement: Supplementary file 1 [file life-13-02346-s001.zip › Table S1-3.pdf]

Table S1A Lifespan assay with neuronal rescue of NHR-49

Strain	Background/Genotype	Trial1				Bonferroni P-value	
		n	Mean (d)	SE+/-	% (vs N2)	P(vs N2)	P(vs nhr-49)
N2	Wild type	99	16.2	0.36	100		
STE68	*nhr-49*	161	9.64	0.16	59.5	<0.0001	
KHY140	*nhr-49;okyEx104[rgef-1p::NHR-49::SL2::GFP]*	109	13.9	0.22	85.8	<0.0001	<0.0001
Trial2							
N2	Wild type	99	16.68	0.33	100		
STE68	*nhr-49*	111	10.65	0.13	63.8	<0.0001	
KHY140	*nhr-49;okyEx104[rgef-1p::NHR-49::SL2::GFP]*	109	13.58	0.21	81.4	<0.0001	<0.0001
Trial3							
N2	Wild type	108	16.76	0.24	100		
STE68	*nhr-49*	111	10.15	0.11	60.6	<0.0001	
KHY140	*nhr-49;okyEx104[rgef-1p::NHR-49::SL2::GFP]*	110	14.98	0.22	89.4	<0.0001	<0.0001

Table S1B Lifespan assay with muscle rescue of NHR-49

Strain	Background/Genotype	Trial1				Bonferroni P-value	
		n	Mean (d)	SE+/-	% (vs N2)	P(vs N2)	P(vs nhr-49)
N2	Wild type	102	15.96	0.33	100		
STE68	*nhr-49*	108	8.31	0.12	52.1	<0.0001	
KHY216	*nhr-49; okyEx109[myo-3p::nhr-49+myo-2p::mCherry]*	104	8.26	0.15	51.8	<0.0001	1
Trial2							
N2	Wild type	106	13.76	0.3	100		
STE68	*nhr-49*	108	8.96	0.12	65.1	<0.0001	
KHY216	*nhr-49; okyEx109[myo-3p::nhr-49+myo-2p::mCherry]*	108	10.16	0.19	73.8	<0.0001	<0.0001
Trial3							
N2	Wild type	110	17.86	0.44	100		
STE68	*nhr-49*	112	9.56	0.1	53.5	<0.0001	
KHY216	*nhr-49; okyEx109[myo-3p::nhr-49+myo-2p::mCherry]*	110	10.24	0.11	57.3	<0.0001	<0.0001

Table S1C Lifespan assay with intestinal rescue of NHR-49

Strain	Background/Genotype	Trial1				Bonferroni P-value	
		n	Mean (d)	SE+/-	% (vs N2)	P(vs N2)	P(vs nhr-49)
N2	Wild type	53	11.77	0.42	100		
STE68	*nhr-49*	54	5.94	0.18	50.5	<0.0001	
WBM409	*nhr-49;wbmEx149[ges-1p::3xHA::nhr-49(cDNA)::unc-54 3' UTR + myo-3p::mCherry::unc-54 3'UTR]*	51	10.27	0.33	87.3	0.0028	<0.0001
Trial2							
N2	Wild type	81	14.99	0.32	100		
STE68	*nhr-49*	54	9.07	0.27	60.5	<0.0001	
WBM409	*nhr-49;wbmEx149[ges-1p::3xHA::nhr-49(cDNA)::unc-54 3' UTR + myo-3p::mCherry::unc-54 3'UTR]*	132	12.93	0.24	86.3	<0.0001	<0.0001
Trial3							
N2	Wild type	111	13.76	0.21	100		
STE68	*nhr-49*	92	5.61	0.11	40.8	<0.0001	
WBM409	*nhr-49;wbmEx149[ges-1p::3xHA::nhr-49(cDNA)::unc-54 3' UTR + myo-3p::mCherry::unc-54 3'UTR]*	111	9.22	0.31	67.0	<0.0001	<0.0001

Underline Trials shown in Figure 1

Table S1D Lifespan assay with cholinergic neuron rescue of NHR-49

Strain	Background/Genotype	Trial1				Bonferroni P-value	
		n	Mean (d)	SE+/-	% (vs N2)	P(vs N2)	P(vs nhr-49)
N2	Wild type	106	18.44	0.36	100		
STE68	*nhr-49*	108	7.57	0.14	41.0	<0.0001	
KHY163	*nhr-49; okyEx112[unc-17p::nhr-49::SL2::gfp]*	113	16.58	0.4	89.9	0.0019	<0.0001
Trial2							
N2	Wild type	108	15.55	0.3	100		
STE68	*nhr-49*	109	7.85	0.13	50.5	<0.0001	
KHY163	*nhr-49; okyEx112[unc-17p::nhr-49::SL2::gfp]*	109	13.4	0.28	86.2	<0.0001	<0.0001
Trial3							
N2	Wild type	104	15.66	0.44	100		
STE68	*nhr-49*	107	9.28	0.11	59.3	<0.0001	
KHY163	*nhr-49; okyEx112[unc-17p::nhr-49::SL2::gfp]*	43	12.91	0.7	82.4	0.0012	<0.0001

Table S1E Lifespan assay with serotonergic neuron rescue of NHR-49

Strain	Background/Genotype	Trial1				Bonferroni P-value	
		n	Mean (d)	SE+/-	% (vs N2)	P(vs N2)	P(vs nhr-49)
N2	Wild type	53	11.77	0.42	100		
STE68	*nhr-49*	52	6.06	0.17	51.5	<0.0001	
KHY179	*nhr-49;okyEx115[tph-1p::NHR-49::SL2::gfp::let-858 3'UTR]*	52	9.13	0.37	77.6	<0.0001	<0.0001
Trial2							
N2	Wild type	108	15.55	0.3	100		
STE68	*nhr-49*	109	7.85	0.13	50.5	<0.0001	
KHY179	*nhr-49;okyEx115[tph-1p::NHR-49::SL2::gfp::let-858 3'UTR]*	83	11.84	0.23	76.1	<0.0001	<0.0001
Trial3							
N2	Wild type	111	13.76	0.21	100		
STE68	*nhr-49*	92	5.61	0.11	40.8	<0.0001	
KHY179	*nhr-49;okyEx115[tph-1p::NHR-49::SL2::gfp::let-858 3'UTR]*	91	9.51	0.32	69.1	<0.0001	<0.0001

Table S1F Lifespan assay with dopaminergic neuron rescue of NHR-49

Strain	Background/Genotype	Trial1				Bonferroni P-value	
		n	Mean (d)	SE+/-	% (vs N2)	P(vs N2)	P(vs nhr-49)
N2	Wild type	108	15.55	0.3	100		
STE68	*nhr-49*	109	7.85	0.13	50.5	<0.0001	
KHY180	*nhr-49; okyEx116[dat-1p::nhr-49::SL2::gfp::let-858 3'UTR]*	110	9.05	0.14	58.2	<0.0001	<0.0001
Trial2							
N2	Wild type	106	18.44	0.36	100		
STE68	*nhr-49*	108	7.57	0.14	41.1	<0.0001	
KHY180	*nhr-49; okyEx116[dat-1p::nhr-49::SL2::gfp::let-858 3'UTR]*	112	8.65	0.19	46.9	<0.0001	<0.0001
Trial3							
N2	Wild type	81	14.99	0.32	100		
STE68	*nhr-49*	54	9.07	0.27	60.5	<0.0001	
KHY180	*nhr-49; okyEx116[dat-1p::nhr-49::SL2::gfp::let-858 3'UTR]*	56	9.5	0.26	63.4	<0.0001	0.5225

Table S1G Lifespan assay with GABAergic neuron rescue of NHR-49

Strain	Background/Genotype	Trial1				Bonferroni P-value	
		n	Mean (d)	SE+/-	% (vs N2)	P(vs N2)	P(vs nhr-49)
N2	Wild type	53	16.43	0.46	100		
STE68	*nhr-49*	52	10.15	0.21	61.8	<0.0001	
KHY153	*nhr-49; unc-25p::nhr-49::SL2::gfp*	112	9.87	0.17	60.0	<0.0001	0.5606
Trial2							
N2	Wild type	111	13.76	0.21	100		
STE68	*nhr-49*	92	5.61	0.11	40.8	<0.0001	
KHY153	*nhr-49; unc-25p::nhr-49::SL2::gfp*	113	6.76	0.16	49.1	<0.0001	<0.0001

Underline Trials shown in Figure 1

Table S2. All strains used for this study

Experimental Models: Organisms/strains	Source	Identifier
nhr-49(nr2041); okyEx104[rgef-1p::nhr-49::SL2::gfp]	This study	KHY140
nhr-49(nr2041); wbmEx149[ges-1p::3xHA::nhr-49(cDNA)::unc-54 3'UTR + myo-3p::mCherry]	CGC	WBM409
nhr-49(nr2041); okyEx109[myo-3p::nhr-49 + myo-2p::mCherry]	This study	KHY216
nhr-49(nr2041); okyEx110[unc-17p::nhr-49::SL2::gfp]	This study	KHY161
nhr-49(nr2041); okyEx114[eat-4p::nhr-49::SL2::gfp]	This study	KHY183
nhr-49(nr2041); okyEx115[tph-1p::nhr-49::SL2::gfp]	This study	KHY179
nhr-49(nr2041); okyEx116[dat-1p::nhr-49::SL2::gfp + unc-122p::gfp]	This study	KHY180
nhr-49(nr2041); okyEx134[unc-25p::nhr-49::SL2::gfp + unc-122p::gfp]	This study	KHY153
egl-6(n592)	CGC	MT14666
nhr-49(nr2041); egl-6(n592)	This study	KHY225

Table S3. All primers used for this study. Restriction enzyme sites are indicated by underline.

Cloning Primers		Forward 5'-3'		Reverse 5'-3'
Prgef-1		attattCTGCAGCGTTTCCGATACCCCCTTATATCAG		aataatGGATCCTTTACTGCTCATCGTCGTCGT
Punc-17 (nested)	in	aatgaaataagcttGCATGCAACGTATACACCAATCATTTCTCCC	in	gaaagtagtccatCCCGGGAGACTTTCGATGAATTACCTGAAAATTA
	out	agcatctcatctcaatctccgg	out	acgggcacgttgaagcccat
Peat-4		aataatGCATGCTCAGTTTGAAATTGCTGCTCT		TGCAGTCATTCTGAAATTAGTTGA
Ptph-1		GCGGCCGCAATAAGTTATTGTACCCTGACCAAAAC		ttCCCGGGATGATTGAAGAGAGCAATGCTAC
Pdat-1		GCGGCCGCCCATGAAATGGAACTTGAATCCAG		ttCCCGGGGGCTAAAAATTGTTGAGATTCGAG
Punc-25		atttttGCATGCAAAAAACACCCACT		aataatCCCGGGTTTTGGCGGTGAACTGAGCTTTTC
nhr-49		tttCCCGGGATGGACTACTTTCTTGAT		aaaGGATCCTTAGAGCATATGATTATTCTG
SL2		aatGGATCCCGCTGTCTCATCCTACTTTC		aatGGTACCAGCAGTTTCCCTGAATTAAAATTAGA
MCS		AGCTTGAATTCGCGGCCGCTCTAGA CCCGGGG		ACTTAACCGCCGGCGAGATCTGGGCCCCCTAG
