# Supplementary figures and images for: Dissecting the Neuronal Contributions of the Lipid Regulator NHR-49 Function in Lifespan and Behavior in C. elegans
Source: Life (Basel). 2023 Dec 15;13(12):2346. doi: 10.3390/life13122346 (PMC10744624; doi:10.3390/life13122346)

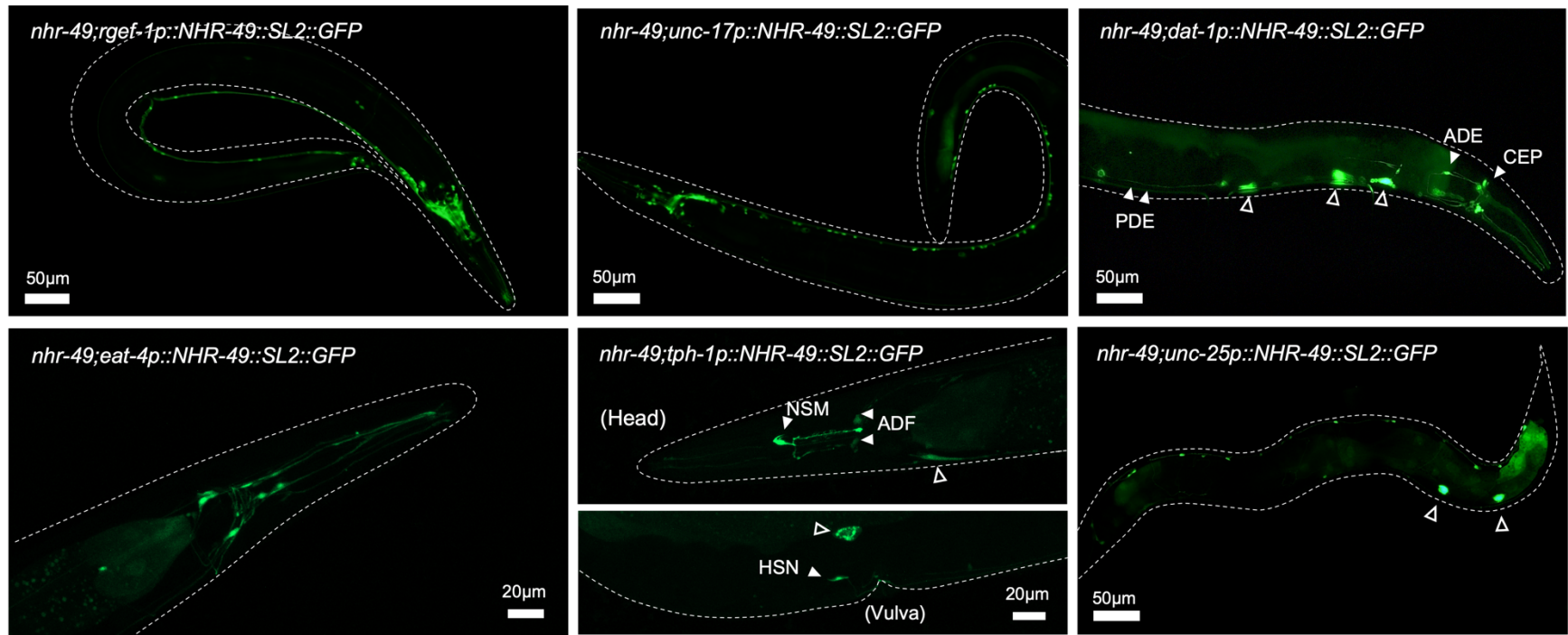

Figure S1

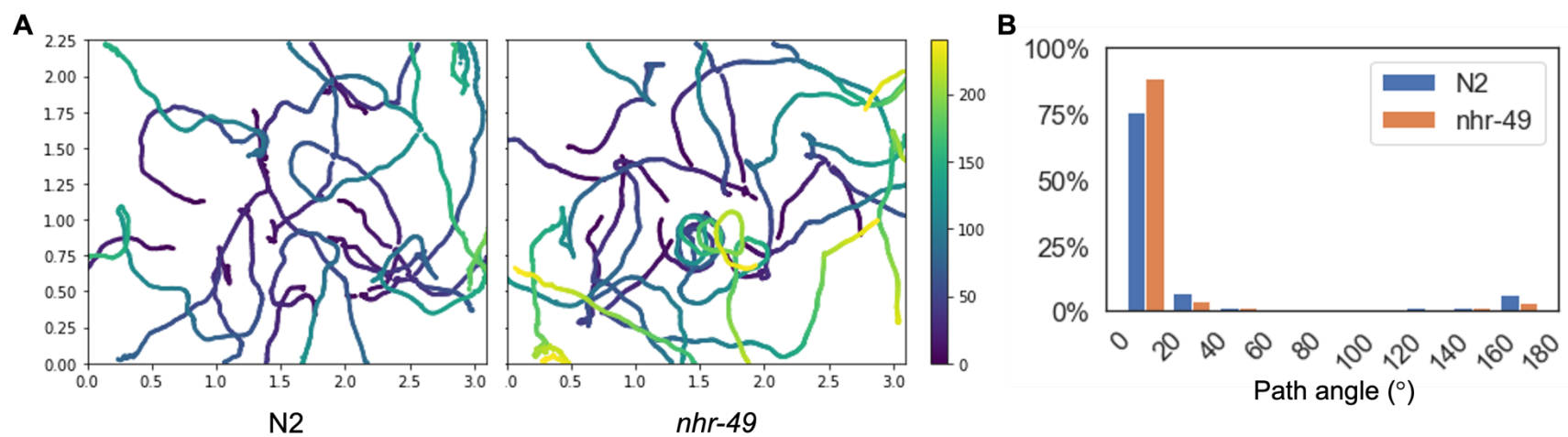

Figure S2

Supplement: Supplementary file 1 [file life-13-02346-s001.zip › SupplementaryFigures.pdf]
